# Trait self-consciousness predicts amygdala activation and its functional brain connectivity during emotional suppression: an fMRI analysis

**DOI:** 10.1038/s41598-017-00073-3

**Published:** 2017-03-08

**Authors:** Shengdong Chen, Changming Chen, Jiemin Yang, Jiajin Yuan

**Affiliations:** 1 The Laboratory for Affect Cognition and Regulation (ACRLAB), Key Laboratory of Cognition and Personality of Ministry of Education (SWU), Faculty of Psychology, Southwest University, Chongqing, China; 2 School of Educational Sciences, Xinyang Normal University, Xinyang, China

## Abstract

The present functional magnetic resonance imaging study investigated how trait neuroticism and its heterogeneous subdimensions are related to the emotional consequences and neural underpinnings of emotion regulation. Two levels of neuroticism assessments were conducted with 47 female subjects, who were required to attend to, suppress emotion displays to, or cognitively reappraise the meanings of negative images. The results showed reduced emotional experience and bilateral amygdala activation during reappraisal, and this regulation effect is unaffected by individual differences in neuroticism and its subdimensions. By contrast, the emotion downregulation effect of suppression in the right amygdala is compromised with increasing self-consciousness but not overall neuroticism dimension. This association holds robust after controlling the potential contribution of habitual suppression. Moreover, the psychophysiological interaction (PPI) analysis revealed that self-consciousness predicts weaker functional coupling of the right amygdala to supplementary motor area and putamen during expressive suppression, two regions mediating the control and execution of motor actions. These findings suggest that self-consciousness predicts increased difficulty in emotional regulation using expressive suppression; and that the heterogeneous nature of trait neuroticism needs to be considered in exploring the association of neuroticism and emotion regulation.

## Introduction

The term emotion regulation refers to those processes that influence the generation, the experience and the expression of emotions^1^. The ability to successfully regulate negative emotions is important for our mental health, well-being and social functions^2, 3^. Conversely, impairments in the ability to regulate negative emotions have been associated with impulsive aggression and violence^4^, mental-health problems^5, 6^ and psychiatric disorders^7^.

The personality trait neuroticism, defined as a predisposition to experience negative affect^8^, is strongly associated with many different mental and physical disorders, such as anxiety disorders, major depression disorders, schizophrenia, and substance use disorders^9–11^. Importantly, individuals with high neuroticism may have a relatively inefficient pattern of emotion regulation, and this may be a risk factor for these mental and physical disorders. Therefore, examining the associations between neuroticism and behavioral and neural indices of emotion regulation is a timely and important research question with high relevance for both basic and clinical research.

A couple of previous studies have used correlational and observational approach to explore this issue. Kokkonen and Pulkkinen^12^ found that trait neuroticism influences the tendencies and attempts to regulate subjective emotion experiences by the mediation of current mood and mood evaluation, irrespective of sex; Ng and Diener^13^ has reported that neuroticism is negatively associated with the tendencies to repair one’s negative emotions. Moreover, it has also been found that neuroticism is negatively associated with the extent of habitual use of cognitive reappraisal^14, 15^. However, what these studies measured are the self-reported attempts, or tendencies of habitual emotional regulation strategies. Currently little is known about the relationship between neuroticism and the regulatory effects of emotion regulation, either at behavioral or neural level.

Therefore, the goal of the present study is to investigate the association between trait neuroticism and neural indices of negative emotion regulation, by manipulating emotion regulation strategies during an fMRI task. In this study, we focused on the two common emotion regulation strategies, cognitive reappraisal and expressive suppression. Cognitive reappraisal involves reinterpretation of the meaning of an emotional situation, whereas expressive suppression involves inhibition of any overt emotion displays (e.g., facial expressions, gestures) and behavioral reactions. In the well-known process model of emotion regulation^1, 16^, the reappraisal and suppression strategies were typical antecedent-focused and response-focused strategies, respectively. The former regulates emotional response tendencies early on, before they give rise to full-fledged responses. By contrast, response-focused strategy works late in the emotion-generative process, by modulating the behavioral output of the emotional reaction, after the emotional response has been fully generated.

Moreover, we are also interested in the influence of subdimensions of neuroticism on regulatory effects of emotion regulation, because that the clinical diagnosis and the assessment of affective disturbances depend more on individual subdimensions than on the overall neuroticism factor^17, 18^, and that subdimension better corresponds to specific physiological system^19^ and behavior^20^ relative to the overall factor. Within big-five personality construct, neuroticism has been considered as a heterogeneous trait consisting of six discrete subdimensions; i.e., anxiety, depression, hostility, self-consciousness, impulsiveness and vulnerability to stress^21–23^. Such subdimensions reflect distinct cognitive and emotional constructs despite low to moderate correlations with each other in factor analyses^22^. In this study, we focused on anxiety, depression and self-consciousness subdimensions that have been suggested to be highly relevant to habitual use of cognitive reappraisal and expressive suppression. Individuals with more symptoms of anxiety and depression reported more frequent use of expressive suppression and less frequent use of cognitive reappraisal^7, 14, 24^. Clinically, people diagnosed with generalized anxiety disorder (GAD) or major depressive disorder (MDD) are both marked by dysfunctional emotion regulation^25–27^. The subdimension self-consciousness is akin to social anxiety^28, 29^, and it has been indicated that negative social emotions of shame and embarrassment form the central components of self-consciousness^30^. Individuals with high self-consciousness, as reflected by higher social anxiety, tend to have greater use of emotional expressive suppression as a way to avoid potential social rejection in comparison with those low in self-consciousness^31–33^.

We used emotionally negative pictures to induce negative emotions, and assessed the negative emotion regulation effects by the activation of bilateral amygdala and amygdala-related functional connectivity during the regulation relative to no-regulation conditions. We concentrated on amygdala because previous studies of emotion regulation have indicated that amygdala-related measures represent key neural underpinnings of the induction and regulation of negative emotion^34–36^. Specifically, the extent of amygdala activation increases during passive viewing of negative stimuli and decreases significantly during emotion down-regulation processes like cognitive reappraisal^37^, or expressive suppression^38^. Furthermore, the greater amygdala-related functional connectivity during cognitive reappraisal has been suggested to be associated with lower intensity of negative affect^39^, whereas less amygdala-related functional connectivity during passive viewing of negative stimuli with depression symptoms^40^. Given that expressive suppression is as effective as reappraisal in reducing immediate emotion impacts in East Asian populations^41, 42^, we predict that negative pictures should induce robust amygdala activity, and the reappraisal or suppression should effectively decrease amygdala activations.

Given the above evidence and the heterogeneous nature of trait neuroticism, we hypothesize that the three subdimensions should predict amygdala activation and amygdala-related functional connectivity better than the overall factor of neuroticism. Specifically, as individuals with increasing anxiety, depression or self-consciousness, are associated with enhanced negative emotion which, in turn, increases with greater daily use of suppression^24, 28, 31^, we predict that the emotion regulation effect of suppression might be compromised, or even reversed in people high in these facets, as manifested by increased amygdala activity and decreased functional connectivity between amygdala and inhibition-related regions during suppression. By contrast, considering the beneficial nature of the reappraisal strategy^14, 31^, it is likely to observe that reappraisal results in reduced amygdala activations, irrespective of individual differences in specific neuroticism facets.

## Results

We listed the results of commonly used parametric test here. Moreover, in order to address the potential problem of the normality assumption, we also used the parametric test to reanalyze our main results (see Supplementary Material). The results of parametric test are identical to of permutation test, showing that our results are not influenced by the use of statistical methods.

### Negative Emotion Induction

#### Subjective Experience

**Figure 1:**
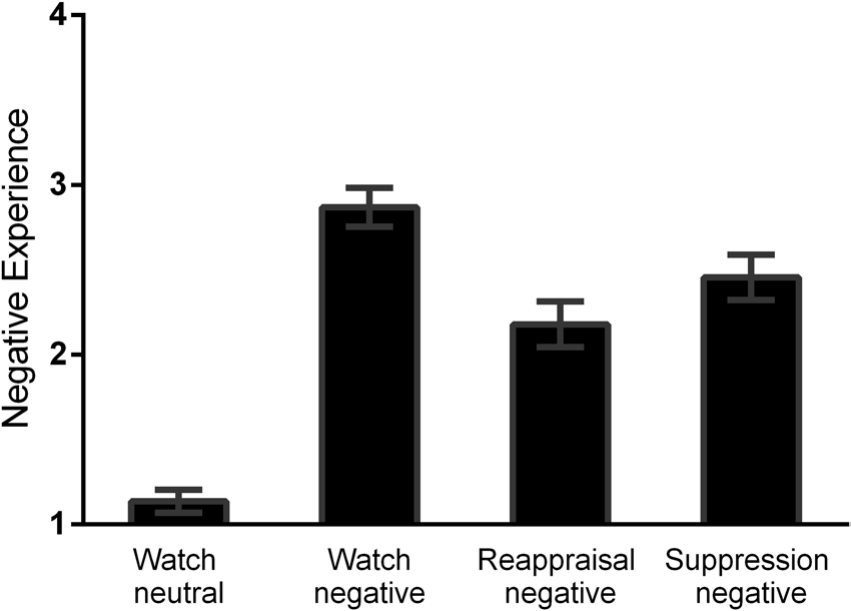
Mean negative emotion ratings during emotion induction and regulation sessions. Error bars = SEM.

Compared to watch-neutral condition, participants experienced significantly (t = 17.65, p < 0.001) more negative emotion under the watch-negative condition (Fig. 1).

#### Amygdala Responses

**Figure 2:**
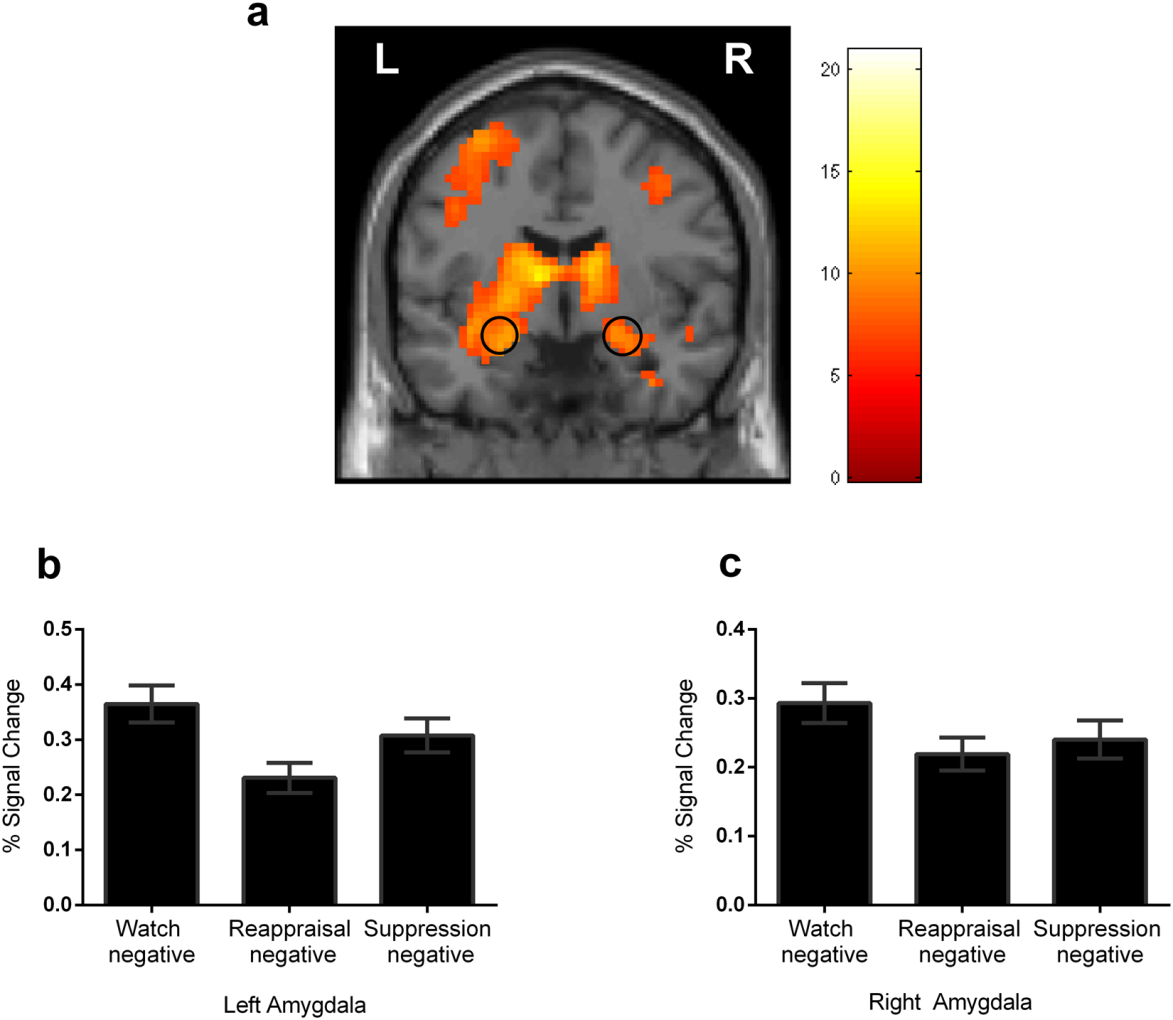
**(a)** Amygdala responses to negative stimuli. From the one-sample t-test across all 48 subjects for the contrast watch-negative > watch-neutral. The display threshold was FWE-corrected p < 0.001 with an extent of ten voxels. Peak of amygdala activation was centered at (t = 9.29, x = 27, y = −3, z = −18) in the right and (t = 10.98, x = −21, y = 0, z = −12) in the left. **(b)** Left amygdala responses during the instructed emotion regulation. **(c)** Right amygdala responses during the instructed emotion regulation. Error bars = SEM.

As expected, the watch-negative versus watch-neutral contrast resulted in enhanced responses in bilateral amygdala (Fig. 2a) and temporal, occipital and parietal cortex, and the other subcortical regions (Table 1).

**Table 1:** Group activations for Contrast Watch-negative Versus Watch-Neutral.

Brain Regions	Brodmann	x	y	z	t-Value
**Watch-negative > Watch-Neutral**					
Temporal and Occipital Lobes					
R Fusiform	37	42	−51	−18	20.87
R Middle Temporal Gyrus	37	51	−72	0	19.26
L Inferior Temporal Gyrus	19	−48	−75	6	17.73
L Middle Occipital Gyrus	19	−48	−78	6	15.40
L Fusiform	19	−39	−51	18	14.86
Parietal Lobes					
Angular Gyrus	7	27	−48	45	8.08
	7	30	−57	51	7.29
Subcortical Regions					
L Hippocampus		−30	−15	−9	14.18
L Caudate		−9	0	12	13.99
L Thalamus		−15	−12	15	13.27
L Amygdala		−21	0	−12	10.98
R Amygdala		27	−3	−18	9.29
**Watch-Neutral > Watch-negative**					
R Precentral Gyrus	4	39	−18	54	8.11
	4	36	−21	63	8.01
L Parahippocampal Gyrus	37	−33	−42	−3	8.67

*Note.* All clusters reached a significance level of p = 0.001, FWE corrected and an extent threshold of 10 voxels. For each cluster, x, y, z, MNI coordinates; L, left; R, right.

### Negative Emotion Regulation Effects

We first analyzed the ratings of the extent to which participants followed the instructions by one-simple tests. The results showed that participants’ ratings across watching, reappraisal and suppression sessions were all significantly higher than 3 (all p < 0.01), indicating that participants complied with the experimental instruction successfully. Given participants’ success in implementing the instructions, we next examined whether reappraisal and suppression down-regulated subjective experience and amygdala responses effectively.

#### Subjective Experience

One-way repeated measures ANOVA revealed a significant main effect of emotion regulation strategy (F(2,92) = 25.1, p < 0.001). Post-hoc comparisons showed significantly less intense emotional experiences during the reappraisal-negative (p < 0.001) and suppression-negative (P < 0.001) than during watch-negative conditions; while emotional experiences were further decreased during reappraisal relative to suppression conditions (p = 0.01) (see Fig. 1).

#### Amygdala Responses

Repeated measurement ANOVA of the PSC in the bilateral ROIs revealed a significant main effect of strategy in both ROIs (left: F(2,92) = 6.75, p = 0.002; right: F(2,92) = 3.91, p = 0.02) (Fig. 2b,c). As expected, post-hoc t-tests (one-tailed) revealed that the PSC was significantly lower during reappraisal (left: p = 0.001; right: p = 0.01) and suppression (left: p = 0.038; right: p = 0.034) relative to watching conditions in bilateral amygdala. And in the left amygdala, we found lower PSC during reappraisal relative to suppression conditions (p = 0.021).

### Relationships between the Regulation Effects and Neuroticism Subdimensions

#### Effects of reappraisal

The results of linear regression analyses showed no significant (p > 0.05) association between the overall neuroticism factor and the effects of reappraisal at bilateral amygdala. The multiple linear regression also showed no significant (p > 0.05) association between subdimensions of neuroticism (anxiety, depression and self-consciousness) and reappraisal effects in the bilateral amygdala.

#### Effects of suppression

**Figure 3:**
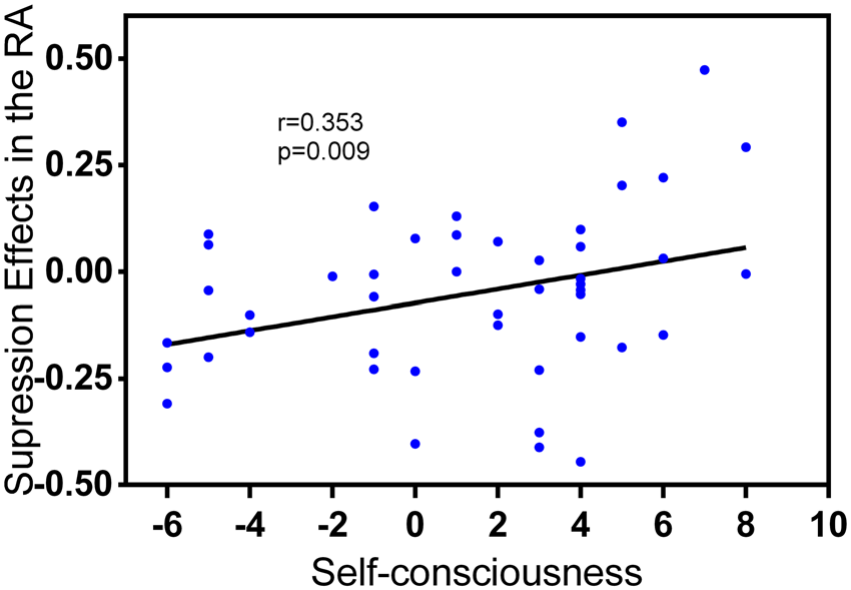
Scatterplot of self-consciousness and the emotion regulation effects of suppression (suppression-watching contrast) in the right amygdala (RA).

The linear regression analysis showed no significant (p > 0.05) association between the overall neuroticism factor and the effects of suppression at bilateral amygdala. However, the analysis of multiple linear regression, with neuroticism subdimensions of anxiety, depression and self-consciousness as predictors, showed that this model fairly significantly accounted for the emotion regulation effects of suppression in the right but not the left amygdala, F(3,43) = 2.69, p = 0.058, with an R^2^ of 15.8% (adjusted R^2^ = 10%). No outliers were found (standard residuals = Std. Residual; Std. Residual Min = −2.24, Std. Residual Max = 2.37), and collinearity diagnostics indicated that multicollinearity was not a concern (anxiety, VIF = 2.04; depression, VIF = 2.32; self-consciousness, VIF = 2.0). However, only trait self-consciousness (β = 0.492, p = 0.017), but not anxiety (β = −0.296, p = 0.146) or depression (β = 0.03, p = 0.9), significantly predicts the emotion regulation effect of suppression in the right amygdala. The regulation effect of suppression, as defined by the reductions of neural activations during suppression relative to watching conditions, was significantly compromised with increasing self-consciousness in the right amygdala. The significant contribution of self-consciousness is still robust after isolating the potential contribution of habitual use of suppression out of the model (r = 0.353, p = 0.009, one-tailed). Besides, in order to ensure that this association not also results from the self-conscious individuals’ increased reactivity to negative stimuli, we examined the relationship between the self-consciousness and the emotion responses in right amygdala (watch negative > watch neutral), and no significant result was found (p = 0.43). The inter-correlations between IVs, DVs and covariables, and the scatterplot for this regression are reported in Table 2 and Fig. 3, respectively.

**Table 2:** Inter-correlations among the neuroticism subdimensions, the emotion regulation effects, and the habitual use of reappraisal and suppression.

	Emotion regulation effects				Emotion regulation strategy	
	LA_Reap	LA_Supp	RA_Reap	RA_Supp	Reappraisal	Suppression
Neuroticism	−0.054	0.061	−0.026	0.089	−0.22	0.333^*^
Anxiety	−0.155	−0.098	−0.067	−0.029	−0.096	0.429^*^
Depression	−0.072	0.139	0.063	0.148	−0.170	0.295^*^
Self-consciousness	−0.056	0.158	0.184	0.330^*^	−0.268^*^	0.292^*^

^*^p < 0.05 level (one-tailed).

The emotion regulation effects were represented by the contrast value: LA_Reap, left amygdala, reappraisal vs. watching; LA_Supp, left amygdala, suppression vs. watching; RA_Reap, right amygdala, reappraisal vs. watching; RA_Supp, right amygdala, suppression vs. watching.

### Functional Connectivity during Expressive Suppression and Relation with Self-consciousness

**Figure 4:**
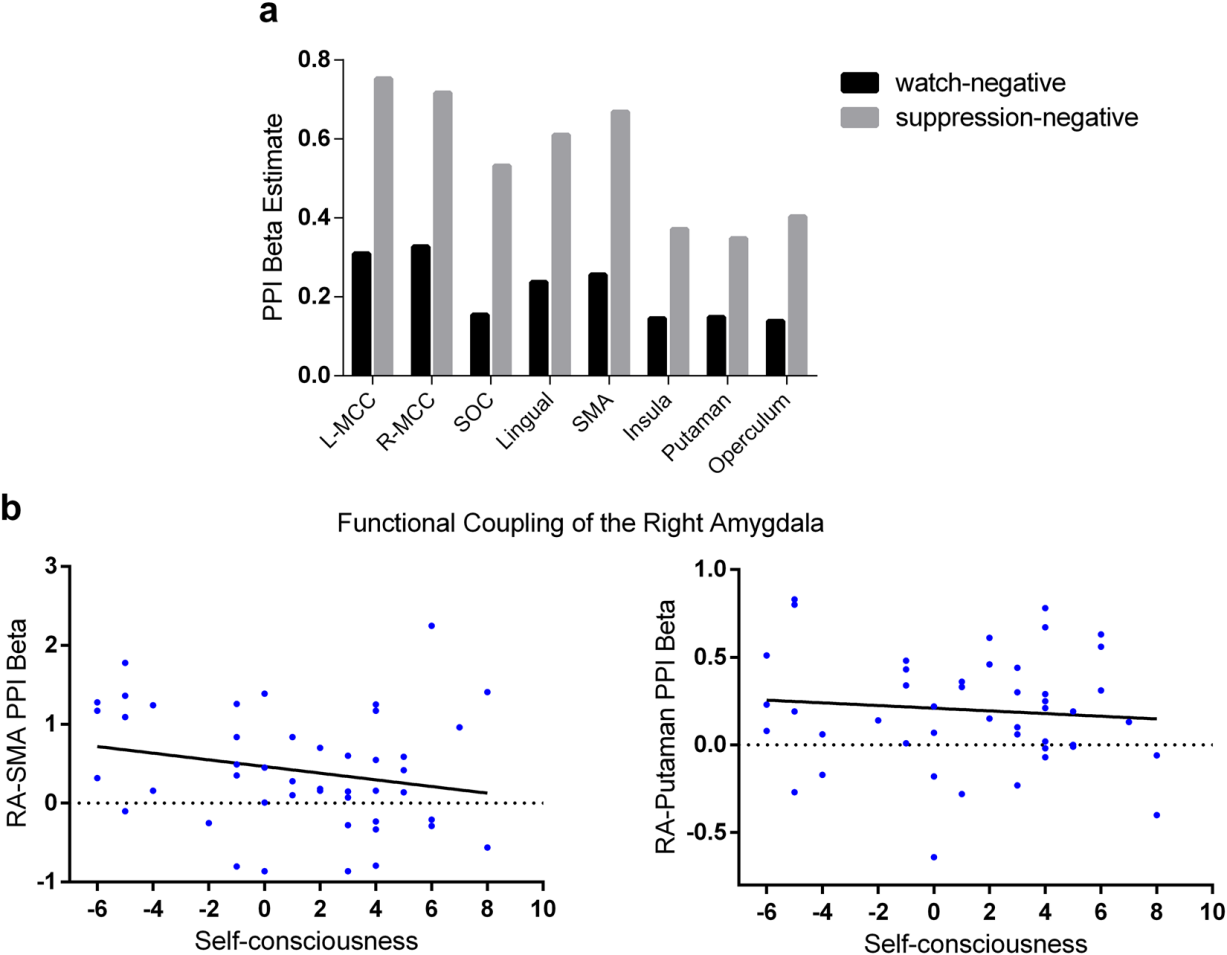
**(a)** The bar graph showing areas that exhibit significant task-dependent (Suppression vs. Watching contrast) coupling with the right amygdala: bilateral middle cingulum cortex (MCC), superior occipital cortex (SOC), lingual cortex, supplementary motor area (SMA), insula, putamen and rolandic operculum. **(b)** Partial correlations between self-consciousness and amygdala-SMA (left: r = −0.253, p = 0.049) and amygdala-putamen (right: r = −0.266, p = 0.041) PPI beta after isolating the effect of trait anxiety, depression and the habitual use of expressive suppression.

Given that self-consciousness was uniquely associated with increased right amygdala activity during suppression, we further explored whether self-consciousness was also associated with decreased functional connectivity during emotion suppression (suppression-negative vs. watch-negative), with the right amygdala as a source region. The covariance in activity between the right amygdala and eight coupled regions (identified in Table 3) was significantly higher during suppression than during watching conditions (Fig. 4). No pattern of coupling was observed in the reverse contrast (watch-negative vs. suppression-negative).

**Table 3:** Task-related PPI analysis of right amygdala seed.

**Brain region of co-activation Supp-neg > Watch-neg**	**Brodmann**	**x**	**y**	**z**	**Peak t-value**	**Size**
L Middle cingulum cortex	24	−3	6	36	4.89	54
L Rolandic operculum	48	−45	−21	21	4.89	23
L Insula	48	−33	−12	12	4.56	31
L Putamen		−18	12	6	4.3	37
R Superior occipital cortex	19	27	−87	27	4.15	29
R Lingual	18	9	−63	−3	3.97	27
L Supplementary motor area	32	−3	3	48	3.92	25
R Middle cingulum cortex	24	3	21	36	3.48	43
**Watch-neg > Suppression-neg**						
	No significant clusters					

Regions showing right amygdala coupling with clusters of 10 or more contiguous voxels (P < 0.001 uncorrected). For each cluster, x, y, z, MNI coordinates; L, left; R, right.

Partial correlation analysis between self-consciousness and the PPI beta estimate of these significant functional connectivity was conducted after controlling for the effect of trait anxiety, depression and the habitual use of expressive suppression. We found that self-consciousness was significantly and negatively associated with amygdala-SMA (Supplementary Motor Area; r = −0.253, p = 0.049) and amygdala-putamen PPI beta (r = −0.266, p = 0.041). However, these results did not survive a FDR correction for multiple comparison.

## Discussion

The current fMRI study aimed at investigating the association between trait neuroticism and neural and behavioral indices of negative emotion regulation. We first conducted a manipulation check to confirm the successful modulation of amygdala responses by reappraisal and suppression. Results indicated that negative pictures induced intense negative emotional experiences and robust amygdala activation, and both reappraisal and suppression strategies reduced subjective emotional intensity and the amygdala activation. These findings are consistent with the increasing evidence that expressive suppression, like cognitive reappraisal, is able to reduce experiential, physiological or social impacts of emotional events^42, 43^, especially in East Asian cultures that encourage the suppression of emotional displays more than western cultures^41, 44, 45^. Specially, reappraisal relative to suppression condition further decreased the left amygdala activations and the negative emotional experiences. These findings suggest that reappraisal is a more adaptive emotion down-regulation strategy than suppression, consistent with abundant existing evidence that reappraisal reduces emotional impacts of negative events without maladaptive physiological or psychological consequences^14, 37, 46^.

Consistent with our prediction, we observed that none of overall neuroticism and its subdimensions significantly related to the emotion regulation effects of reappraisal in behavioral or amygdala activation measures in this study. This suggests that the regulatory effects of cognitive reappraisal were stable and unaffected by individual differences in trait neuroticism and its subdimensional measures, and that high neurotic individuals should use a more adaptive emotion regulation strategy (e.g., reappraisal) for the successful regulation of their emotions^47^. Moreover, consistent with a recent fMRI study reporting that expressive suppression down-regulated the social-related activation in right amygdala to a larger extent than reappraisal^36^, we found that self-consciousness uniquely predicts reduced emotion regulation effect of expressive suppression in right amygdala activations, confirming that specific subdimensional traits are often closer to specific physiological systems than the overall personality dimension^19^. This association remains robustly existent, even after controlling the potential contribution of habitual use of suppression. As indicated by prior studies, self-consciousness is a trait with close relevance to social anxiety^30–32^. That is, high self-conscious individuals, compared with low self-conscious individuals, tend to have more negative social evaluations^48^, to experience more embarrassed and anxious affects^49^, and to show increased amygdala reactivity^50^ during ambiguous and embarrassing social situations. The negative social evaluations may drive high self -conscious individuals to avoid expressing their emotions to others in order to prevent peer ridicule. However, our findings suggest that expressive suppression is relatively maladaptive for them to downregulate their own negative emotions. Besides, our study also found that self-consciousness is positively correlated with habitual use of suppression (see Table 2), confirming previous findings that individuals with high self-consciousness tend to show more daily use of this strategy^31, 32^. In sum, for people with high self-consciousness, expressive suppression may lead to a worse emotion regulation effect, but they appear to more frequently use this strategy to minimize social evaluation concerns. Future studies should continue to explore the underlying reasons.

However, we observed no such association between the trait anxiety (or depression) and the emotion regulation effects of suppression. One possible reason is that expressive suppression is a social relation-related emotion regulation strategy, whose instruction involves inhibition of one’s overt emotional displays, hiding one’s emotion from being detected by somebody else in the interaction^16, 37^. However, what the measure of trait anxiety or depression reflects is stable emotional state, not necessarily involving interpersonal or social relations. By contrast, the measure of self-consciousness is directly related to social emotions like social anxiety, embarrassment and shame^29, 30^, and individual differences in self-consciousness are directly related to the daily use of expressive suppression as observed in the present study (see Table 2). This might account for why self consciousness but not trait anxiety or depression is significantly related to the emotion regulation effect of expressive suppression in this study.

Moreover, we found that task-dependent (suppression vs. watching) amygdala-putamen/SMA functional connectivity correlated negatively with self-consciousness scores. However, these findings should be treated as exploratory due to being uncorrected for multiple comparisons. The SMA has been suggested to have a role in the planning and monitoring of affective motor behavior^51, 52^, and to be active during expressive suppression^36, 38^. And the putamen, as a key region of basal ganglia, has been shown to be involved in motor performance^53^ and motor preparation^54^. Clinical studies have also found that patients with Parkinson’s disease (PD) consistently show reduced activity of the SMA and putamen^55, 56^. Taken together, the weaker amygdala-putamen/SMA connectivity with increasing self-consciousness might reflect a disconnection between the central areas underlying affective responding and the areas involved in the plan and inhibition of behavioral reactions to an affective stimulus. Given that more self-conscious individuals tend to have increased amygdala activation during expressive suppression relative to watching conditions (Fig. 3), this disconnection might be explained as a less efficient top-down modulation of amygdala activations from motor control-related cortical regions such as SMA.

Several limitations should be acknowledged when considering these findings. First, to maximize negative reactivity and facilitate observation of emotion regulation effects, this study sample included only female subjects as women are known for enhanced emotional susceptibility to negative stimuli^57^. However, there is also evidence showing that women have greater preferences for close interpersonal relation than men, such as higher levels of social involvement^58^ and more relationship-oriented vocational interests^59^. Thus, including female subjects may boost the association between self-consciousness and the emotion regulation effects of suppression. In this regard, whether the current findings can be generalized to male subjects remains undetermined, awaiting future studies for direct examination. Second, though neuroticism broadly refers to an individual’s tendency to experience negative affect, the influence of this disposition on individuals’ emotional responses interacts with situations. It has been indicated that neuroticism is related to negative affect under threat, but not under reward conditions^60, 61^. Thus, future studies also need to assess the association between neuroticism facets and positive emotion regulation of suppression.

In conclusion, we observed that cognitive reappraisal significantly reduced the emotion effects in emotional experiences and amygdala activity, and its emotion regulation effect is unaffected by individual differences in neuroticism and its subdimensional facets. By contrast, though expressive suppression also resulted in reduced emotional experiences and the amygdala activation; self-consciousness, a subdimension of neuroticism, is associated with decreasing emotion regulation effects in the right amygdala, and weaker amygdala to supplementary motor area/putamen functional coupling during expressive suppression relative to watching conditions. These results suggest that increasing self-consciousness is linked with decreased emotion regulation effects of expressive suppression, and self-conscious individuals should use more adaptive strategies, such as reappraisal, to regulate their emotional reactions more effectively.

## Methods

### Participants

Fifty-two healthy, right-handed women (M = 21.0; SD = 1.4 years) were studied, however, forty-seven subjects were submitted to final analysis. Three subjects during menstrual period were excluded because previous research suggests that emotion regulation in women is affected by menstrual cycle^62^. Two participants were excluded for their head movement out of limitation (>3 mm). All participants were provided written informed consent and consented to participate in the study. This study was approved by the local ethical committee of Southwest University and the Institutional Human Participants Review Board of the Southwest University Imaging Center for human brain research. The experimental procedure was in accordance with the ethical principles of the 1964 Declaration of Helsinki^63^. Female subjects were chosen because they show stronger negative emotional responses than men^64, 65^.

### Individual Difference Measures

We administered individual difference measures before training procedure. Participants completed anxiety, depression and self-consciousness and the other three emotional stability measures (8 items for each subdimension) by the Chinese-version of a 48-item Neuroticism domain scale of the Revised Neuroticism Extraversion Openness Personality Inventory (NEO-PI-R)^17^ Six facet scores and a total score are computed. The items were scored on a 5-point scale (from −2 to 2) ranging from ‘strongly disagree’ to ‘strongly agree’. The internal consistencies (*Cronbach alpha*) of neuroticism are 0.65. In addition, individual differences in the habitual use of reappraisal and suppression were controlled by measuring Emotion Regulation Questionnaire, ERQ^14^. Neuroticism and two subdimensions (anxiety and depression) were normally distributed according to the Shapiro–Wilk test (all p > 0.05). Self-consciousness was not strictly normally distributed at the significance level α = 0.05 (p = 0.024). Therefore, in order to avoid the potential problem of the normality assumption, we used the parametric test to check whether our main results were influenced by the use of statistical method (see Supplementary Material).

### Stimuli and Training Procedure

The current study adopted a block-design to present negative and neutral pictures selected from Chinese Affective Picture System^66^, a native affective system adapted from the International Affective Picture System (IAPS) to avoid the cultural bias of emotion inducement in Chinese subjects. Like many other studies using IAPS or CAPS^64, 67, 68^, the pictures used in the present study covered a variety of contents, such as emotionally negative or neutral animals (e.g. snakes, eagles), natural scenes (e.g. natural disasters, clouds) or human activity (e.g. fighting, sports). The validity of the pictures for inducing negative emotion, and the control of emotional attributes across watch, suppression and reappraisal conditions were verified by the subjects’ emotional assessment (see Table 4

**Table 4:** ANOVA and paired results t-test for rating of the valence and arousal of the stimuli.

	Valence			F	p	Arousal			F	p
	Watch	Reap	Supp			Watch	Reap	Supp		
Negative	3.05	3.18	3.04	1.17	0.32	6.45	6.42	6.58	1.43	0.25
Neutral	5.82	5.86	6.03	2.34	0.10	3.50	3.56	3.46	0.40	0.67
t	26.26**	27.78**	21.27**			26.26**	29.7**	27.95**		

Two tailed **p < 0.001.

). All the pictures were identical in size and resolution (7.4 cm × 5.4 cm, 72 pixels/in.), and presented in a rectangular frame (8 cm × 8 cm, 72 pixels/in). During fMRI scanning, these pictures were projected to a screen 6 inches from the participant’s eyes inside the head coil.

Reap, reappraisal condition; Supp, suppression condition. On a 1–9 scale, participants rated the valence (1, very unpleasant; 9, very pleasant) and arousal (1, very calm; 9, very excited) of the stimuli.

Before scanning, participants were trained in a practice session to understand the reappraisal and suppression strategies until they used the strategies successfully. Reappraisal instructions required participants to reappraise the pictures by assuming that the pictures were produced by Photoshop software and focusing on their technical details. Suppression instructions trained participants to keep their face still while viewing pictures so that someone watching their face would not be able to detect what was being experienced subjectively. At the end of each training program, participants were required to rate the extent to which they followed the instructions by a four-point grading scale (1, complete failure; 4, complete success).

### fMRI Task Design

The aim of this paradigm was to assess activity changes in amygdala during emotion regulation. Subjects completed three 8-minute runs to reappraise, suppress, or watch the pictures. Watch instructions encouraged participants to view the pictures attentively and experience the emotions freely if generated. Reappraisal and suppression instructions are similar as described in training procedure. The passive watching session was not counterbalanced with the other two emotion regulation sessions in an attempt to avoid any contamination of the watching session by regulation, and the order of the two regulation sessions was counterbalanced across participants. Each run contained 10 negative blocks and 10 neutral blocks, counterbalanced to control for the order. Each block consisted of 4 negative or 4 neutral pictures and each picture was presented for 2.5 s. Each picture was presented only once to avoid participants’ habituation or anticipation. Following each block, a question ‘How negative do you feel?’ appeared on the screen for 2 s, and participants rated their current emotional state on a four-point scale using the index and middle fingers of both hands on a four button MRI-compatible response box (1 = ‘neutral, emotionless’, up to 4 = ‘extremely negative’). Blocks were separated by a 10 s rest period to minimize emotional/task demand carryover among blocks^69^. After completing the task, participants were also required to rate the extent to which they followed the instructions by a four-point grading scale (1, complete failure; 4, complete success). After scanning, participants rated the valence (1, very unhappy; 9, very happy) and arousal (1, very calm; 9, very excited) of the stimuli by a 1–9 scale outside the scanner.

### fMRI Data Acquisition and Preprocessing

Participants were scanned with a 3 Tesla (Magnetom Trio, Siemens, Erlangen, Germany) scanner. Foam cushions were used to reduce head movements and scanner noise. Whole brain blood oxygenation-level dependent (BOLD) functional images were collected with a gradient echo planar imaging sequence (TR = 2 s; TE = 30 ms; flip angle = 90°; matrix size = 64 × 64; FoV = 22 cm^2^; voxel size = 3.4 × 3.4 × 3 mm^3^). Each functional run contains 225 brain volumes, and each volume comprised 32 axial slices. T1-weighted anatomical image were recorded with a total of 176 slices at a thickness of 1 mm and in-plane resolution of 0.98 × 0.98 mm (TR = 1900 ms; TE = 2.52 ms; flip angle = 9°, matrix = 64 × 64, FoV = 22 cm^2^). Stimulus presentation and behavioral data acquisition were controlled using E-prime software.

The first 5 volumes were discarded. Preprocessing of the fMRI data was conducted using DPABI^70^ and comprised slice-timing, spatial realignment to correct for head movement during the scanning, and nonlinear warping into Montreal Neurologic Institute (MNI) space using unified segmentation on T1 image^71^. Normalized functional images were re-sampled to 3 × 3 × 3 mm voxels and spatially smoothed with a Gaussian Kernel of 6-mm FWHM. Head movement estimates derived from the realignment step were included as regressors in all analyses to help diminish the impact of any movement-related effects on the results.

### fMRI Data Statistical Analysis

The analysis of fMRI data was performed using statistical parametric mapping (SPM8; www.fil.ion.ucl.ac.uk/spm), and custom-written programs in Matlab. For each subject, a voxel-wise whole brain analysis was implemented using the general linear model (GLM). Six periods of interest (watch-negative, watch-neutral, reappraise-negative, reappraise-neutral, suppress-negative and suppress-neutral) were included in the model to compute for linear contrast maps, and six head-motion parameters were included as regressors of no interest to account for head motion effects. We than conducted group level, random-effects analyses for the contrast watch-negative versus watch-neutral to check whether negative pictures activated amygdala responses. The whole brain regression analyses employed a significance threshold of p < 0.001 (FWE corrected) with a ten voxel extent threshold.

Given our prior hypothesis regarding the associations between neuroticism and amygdala activity during emotion regulation, we performed small volume corrections within anatomical mask of the amygdala based on Anatomical Automatic Labeling^72^ by WFU_PickAtlas^73^. A significance threshold (p < 0.001, uncorrected; cluster size > 5) was applied within the AAL amygdala mask at the group level for the contrast watch-negative versus watch-neutral^74^. The threshold was determined via Monte Carlo simulations using Analysis of Functional Neuroimages (AFNI) Program AlphaSim that P < 0.001 and cluster size > 5 voxels corresponded to a corrected P < 0.01. The amygdala voxels reaching the threshold were regarded as specific to emotion responses, and thus defined as a functional region of interest (ROI). Each individual’s mean percent signal change (PSC) in the functional-defined ROI was extracted using Marsbar^75^, calculated and entered into a repeated measure ANOVA. The emotion down-regulation effects were represented using the PSC contrast values (reappraisal-negative vs. watch-negative & suppression-negative vs. watch-negative). The univariate and multiple linear regression analysis were next performed to examine the relationship between the two levels of independent variables (IV; one domain and three subdimensional facets of neuroticism) and the dependent variables (DV; the emotion down-regulation effects in the bilateral amygdala), respectively. We used the variance inflation factor (VIF) as a diagnostic measure of multicollinearity, with values exceeding 10 indicating problematic multicollinearity^76^.

A generalized psychophysiological interaction analysis (gPPI)^77^ was further conducted in order to explore how the functional connectivity^78^ between brain regions varies with experimental conditions. As the significant regulation effect of suppression and its association with neuroticism facets were observed in the right instead of left amygdala, and the emotion regulation effect of reappraisal did not vary with neuroticism measures, the PPI analysis was focused on the suppression versus watching conditions with the right amygdala as the seed region. The time series from this seed were extracted for each subject and deconvolved to obtain an estimate of the neural activity. The condition onset times for negative and neutral blocks during watching and suppression conditions were separately convolved with hemodynamic response function (HRF) to create task (psychological) regressors for each experimental condition. The product of this estimated neuronal time-series and vectors representing each of the onsets for the negative and neutral blocks during watching and suppression conditions was computed. These four interaction terms were then reconvolved with hemodynamic response function (HRF) and entered into a new GLM along with the vectors for the onsets for each condition, the estimated mean time-series and covariates of no interest (i.e. a session mean and six movement parameters derived from realignment corrections). The regression coefficient for these interaction term provides a measure of PPI; a correlation in activity between the seed region and the identified regions that significantly varies across suppression versus watching conditions yields a significant PPI effect. The individual contrast images were then entered into a 2nd-level random analysis, in which task-dependent PPI effects (suppression-negative vs. watch-negative; watch-negative vs. suppression-negative) were investigated using one-sample t-tests (df = 47). We report activation using a threshold of uncorrected p < 0.001 (t > 3.99) with at least 10 contiguous voxels. The PPI beta estimate, a measure of the strength of functional coupling between the right amygdala and each coupled region, was then extracted and used in the subsequent partial correlation analysis.

## Electronic supplementary material

Supplementary information
