# Supplementary material for: Trait self-consciousness predicts amygdala activation and its functional brain connectivity during emotional suppression: an fMRI analysis
Source: Sci Rep. 2017 Mar 8;7:117. doi: 10.1038/s41598-017-00073-3 (PMC5428331; doi:10.1038/s41598-017-00073-3)
Supplement: Supplementary file 1 — Supplementary information [file 41598_2017_73_MOESM1_ESM.pdf]

SUPPLEMENTARY MATERIALS

Trait self-consciousness predicts amygdala activation and its functional brain connectivity during emotional suppression: an fMRI analysis

Shengdong **Chen**[a1]**,** Changming **Chen**[ba1]**,** Jiemin **Yang**[a]**, and** Jiajin **Yuan**[a]

[a]*The Laboratory for Affect Cognition and Regulation (ACRLAB), Key Laboratory of Cognition and Personality of Ministry of Education (SWU), Faculty of Psychology, Southwest University, Chongqing, China*

[b]*School of Educational Sciences, Xinyang Normal University, Xinyang, China*

SUPPLEMENTARY METHODS

In order to avoid the potential problem of the normality assumption and multiple comparisons, we used permutation tests to reanalyze our main results. We conducted the permutation tests by Matlab functions. Specifically, we used the Resampling statistical toolkit to examine the emotion down-regulation effects in amygdala (https://www.mathworks.com/matlabcentral/fileexchange/27960-resampling-statistical-toolkit/content/statistics/statcond.m), and used the R package lmPerm (https://CRAN.R-project.org/package=lmPerm) to examine the associations between the regulatory effects of reappraisal or suppression in amygdala and neuroticism or its sub-dimensions. Number of permutations used to estimate the distribution of the null hypothesis was 1000 for an alpha level of 0.05 [1].

SUPPLEMENTARY RESULTS

Negative Emotion Induction

Subjective Experience. Compared to watch-neutral condition, participants experienced significantly ($t = 17.65$, $p<0.001$) more negative emotion under the watch-negative condition.

Note that the analysis of amygdala responses during the contrast watch-negative versus watch-neutral was performed by a whole brain analysis, and thus was not presented here.

Negative Emotion Regulation Effects

Subjective Experience. One-way repeated measures ANOVA revealed a significant main effect of emotion regulation strategy ($F(2,92)=25.11$, $p<0.0005$. Post-hoc comparisons showed significantly less intense emotional experiences during the reappraisal-negative ($p<0.001$) and suppression-negative ($P<0.001$) than during watch-negative conditions; while emotional

experiences were further decreased during reappraisal relative to suppression conditions ($p=0.007$).

Amygdala Responses. Repeated measurement ANOVA by permutation test of the PSC in the bilateral ROIs revealed a significant main effect of strategy in both ROIs (left: $F(2,92)=6.745$, $p=0.0005$; right: $F(2,92)=3.912$, $p=0.0025$). Post-hoc t-tests (one-tailed) revealed that the PSC was significantly lower during reappraisal (left: $p=0.0035$; right: $p=0.0065$) and suppression (left: $p=.043$; right: $p=.033$) relative to watching conditions in bilateral amygdala.

Relationships between the Regulation Effects and Neuroticism Subdimensions.

Effects of reappraisal. The results of linear regression analyses showed no significant ($p>0.05$) association between the overall neuroticism factor and the effects of reappraisal at bilateral amygdala. The multiple linear regression also showed no significant ($p>0.05$) association between subdimensions of neuroticism (anxiety, depression and self-consciousness) and reappraisal effects in the bilateral amygdala.

Effects of suppression. The linear regression analysis showed no significant ($p>0.05$) association between the overall neuroticism factor and the effects of suppression at bilateral amygdala. However, the analysis of multiple linear regression, with neuroticism subdimensions of anxiety, depression and self-consciousness as predictors, showed that this model fairly significantly accounted for the emotion regulation effects of suppression in the right but not the left amygdala, $F(3,43)=2.693$， $p=0.0578$, with an R^2 of 15.82% (adjusted R^2=9.9%). Residual standard error: 0.1854 on 43 degrees of freedom. However, only trait self-consciousness ($\beta=0.024$, $p=0.019$), but not anxiety ($\beta=-0.012$, $p=0.063$) or depression ($\beta=0.001$, $p=0.82$), significantly predicts the emotion regulation effect of suppression in the right amygdala. It should be noted

that the beta coefficient estimated by lmPerm was unstandardized, while the beta coefficient estimated by SPSS was standardized.

SUPPLEMENTARY CONCLUSION

The results of parametric test are identical to of permutation test, showing that our results are not influenced by the use of statistical methods.

References

1. Manly, B.F.J. Randomization, Bootstrap, and Monte Carlo Methods in Biology. 2nd ed. Chapman and Hall, London (1997).
